# Imagining Simpler Worlds to Understand the Complexity of Our Own

**DOI:** 10.1029/2019MS001753

**Published:** 2019-09-13

**Authors:** Edwin P. Gerber, Kevin DallaSanta, Aman Gupta

**Affiliations:** ^1^ Courant Institute of Mathematical Sciences New York University New York NY USA

## Abstract

The atmospheric circulation response to global warming is important for accurate prediction of climate change on regional scales. For the midlatitudes, shifts in the extratropical jet streams have important consequences for precipitation, blocking, and extreme events. It has proven to be a challenge, however, to predict. For example, the North Atlantic jet stream plays a vital role in the climate of eastern North America and Europe; in the last intercomparison of state‐of‐the‐art climate models, the models did not even agree on the sign of its wintertime response to global warming. Perhaps this should not come as a surprise, as we also lack a comprehensive theory for the impact of warming on the midlatitude circulation. In a recent study, Tan et al. (2019, https://doi.org/10.1029/2018MS001492) constructed models of simpler atmospheres to explore the response of the midlatitude jet to global warming. Their idealized atmospheres highlight the difficulty of developing a comprehensive theory for the midlatitude circulation but also provide pathways to improve models of Earth's atmosphere. Models of simpler atmospheres allow one to isolate the impact of specific atmospheric processes and connect theoretical understanding with comprehensive climate prediction systems. Such models can also be used to explore very different atmospheric regimes, from Earth's past to distant planets.

## Key Points

- The atmospheric circulation response to global warming is both a challenge to predict and to understand
- Models of idealized atmospheres allow a process‐oriented investigation of the circulation response
- A growing number of models of simpler atmospheres are being developed and shared

## 1 A Long Time Ago, in a Galaxy Far, Far Away⋯

Four planets orbit stars just like our own Sun. They are the same size as Earth, with similar orbits and rotation rates, but since their axes of rotation are not tilted, they have no seasonal cycle. Their surfaces are remarkably uniform, “swamp worlds” of fixed heat capacity that cannot transport heat like our ocean, with no topographic features or differences in albedo to perturb the zonal structure of the climate. The atmospheric composition on each planet varies, however, leading to different interactions between their atmospheres and outgoing terrestrial (infrared) radiation.

On the first planet, the atmosphere interacts uniformly with all infrared frequencies. Think of it as “gray radiation” (e.g., Frierson et al., 2006), in that radiative transfer—absorption, scattering, and emission—does not depend on the color or frequency of the light. On Earth, the key players in the infrared, water vapor and carbon dioxide, only interact with certain frequency bands due to their molecular structure, which has a significant impact on how radiative heating and cooling are distributed throughout the atmospheric column.

The overall opacity, or thickness of the atmosphere to radiation, is comparable to Earth's atmosphere but exhibits no temporal variation. This is because even though the planet has the equivalent of a hydrological cycle, its condensible “water vapor” is transparent to radiation, and there are no clouds, which would interact with both the outgoing terrestrial and the incoming solar radiation. The circulation can thus transport latent energy upward and polarward, influencing both the atmospheric stability and equator‐to‐pole temperature gradients, but the water vapor itself has no direct interaction with radiative transfer.

On the second planet, radiative transfer depends on the frequency of the terrestrial radiation. As a result, the distribution of radiative heating and cooling is more similar to that on Earth, notably in differences between the troposphere and stratosphere. Frequencies with which the atmosphere interacts weakly allow radiation from the surface and lower troposphere to more easily escape to space. To maintain energy balance, the radiation escaping at frequencies that are more strongly absorbed and emitted must be reduced. This requires cooling at upper levels, where only these frequencies can still interact with the thinning atmosphere. The opacity is still constant in time, however, because there is no direct interaction between infrared radiation and the planet's water vapor.

The third planet differs in that its water vapor can absorb and emit infrared radiation, albeit more simply than on Earth. The hydrological cycle now directly interacts with radiative transfer, allowing for a feedback between water vapor and temperature. When the atmosphere holds more moisture, it becomes more optically thick, trapping infrared radiation that warms the surface and lower atmosphere until the atmosphere can come back into radiative balance. A feedback loop is possible, as warmer air can in turn hold more moisture. The circulation, however, still plays a key role in the actual water vapor content, regulating the strength of the feedback.

Finally, the fourth world is the most like our Earth, with fully interactive radiative transfer and “real” water vapor. Radiative heating on this planet is like that on Earth, except that there are no clouds. In our atmosphere, clouds have a net cooling impact on the extratropics, as their ability to cool the planet by reflecting solar radiation outweighs their tendency to warm it by trapping terrestrial radiation. The surface albedo of the fourth planet is a bit higher than on Earth, such that it still has the same mean surface temperature and hence a comparable amount of moisture in the atmosphere.

How would global warming impact the atmospheric circulation on these planets? What would you expect to happen to the midlatitude jet streams and associated storm tracks? It sounds much easier than predicting climate change on Earth: The surface is much simpler, there is no seasonal cycle, and no coupled atmosphere‐ocean variability. Furthermore, there are no cloud, aerosol, or atmospheric chemistry feedbacks. The answer, however, is still far from trivial.

As explored by Tan et al. (2019), the circulation would respond rather differently on each planet. On the first, with the gray atmosphere, the midlatitude jet streams and storm tracks would initially shift equatorward in response to enhanced greenhouse gas concentrations. This is not what we expect to happen on Earth, where our models (generally) indicate the jet streams and associated midlatitude storms should move poleward.

On the other planets, the jets and storm tracks would expand poleward, albeit at very different rates. The circulation response becomes more sensitive to surface temperature as the transfer of longwave radiation through the atmosphere becomes more Earth‐like, in terms of both the vertical structure of the radiative heating/cooling and its ability to interact with water vapor.

## 2 The Challenge of Midlatitude Circulation Trends

Tan et al. (2019) were not motivated by recent discoveries of exoplanets: Understanding the response of the storm tracks on these hypothetical worlds has direct relevance to climate change on Earth. We have little confidence in our climate model projections of the atmospheric circulation response to global warming, particularly for the midlatitude jet streams and storm tracks (e.g., Shepherd, 2014; Vallis et al., 2015).

This is a big problem for regional climate prediction: We know from observations that shifts in the jet stream impact precipitation and extreme events (e.g., Thompson & Wallace, 2001). Our regional climate models can capture these changes but depend on global climate models for an accurate prediction of the incoming wind and moisture conditions. If you want to predict the local weather, “the answer, my friend, is blowin' in the wind.”

**Figure 1:**
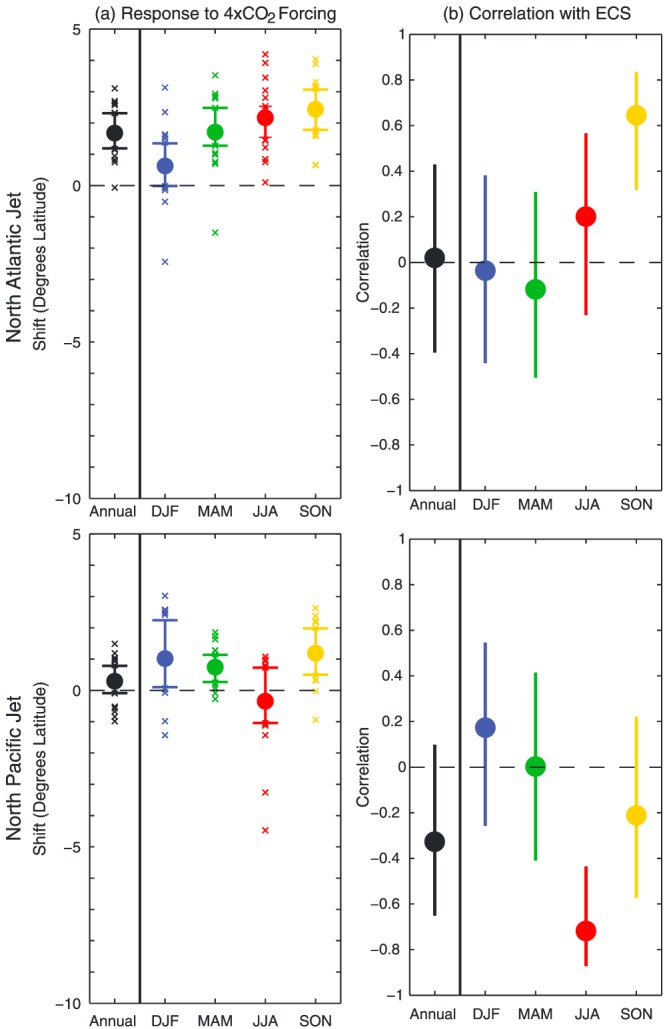
The response of the jets in the (top) North Atlantic sector (300–360°E) and (bottom) North Pacific sector (135–235°E) to 4xCO_2_ forcing in Coupled Model Intercomparison Project, Phase 5 climate models. (a) The multimodel mean (solid dot), 25th–75th percentile range (horizontal bars), and individual model (small x marks) responses, both for the annual mean and the four seasons. (b) The correlation between the intermodel spread in the jet response and the models' Equilibrium Climate Sensitivity (ECS, the equilibrium temperature response to double CO_2_ forcing), with a 95% error bound marked by vertical lines. It is only for autumn in the North Atlantic, and summer in the North Pacific, when there exists a statistically significant increase in the magnitude of the jet shift in models that warm more in response to CO_2_ increase. Reproduced from Grise and Polvani (2016), their Figure 9. DJF = December‐January‐February; MAM = March‐April‐May; JJA = June‐July‐August; SON = September‐October‐November.

Based on most climate model simulations of global warming, we expect the midlatitude jet streams and storm tracks to shift poleward in response to increased greenhouse gas concentrations (e.g., Yin, 2005). When one considers the details, however, the situation quickly becomes murky. For example, in the Coupled Model Intercomparison Project 5 models, there is a great deal of spread in the response of the Pacific and Atlantic storm tracks (e.g., Barnes & Polvani, 2013). As shown in Figure 1a, the response of the jets in Coupled Model Intercomparison Project 5 models to quadrupled CO_2_ varies substantially in different seasons and between the North Atlantic and the North Pacific (Grise & Polvani, 2016). Furthermore, Figure 1b shows that models that warm more in response to greenhouse gas forcing do not necessarily exhibit stronger circulation trends. Thus, narrowing down the climate sensitivity of our planet may not necessarily improve our ability to project circulation changes.

## 3 A Focus on Atmospheric Processes

The difficulty in simulating midlatitude circulation trends mirrors—or perhaps stems from—a lack of understanding. There is not a generally accepted theory that predicts whether the extratropical jets should move poleward or equatorward in response to global warming, let alone the amplitude of the response. At a workshop on storm tracks held near Stockholm last summer (http://climdyn.misu.su.se/stormtracks2018/), roughly a dozen theories were discussed, most of them equally plausible (or at least equally unfalsifiable) given the range of change we have observed.

Tan et al. (2019) help explain why a theory may be so elusive: The response of the circulation to warming differs qualitatively depending on how radiative transfer distributes heating and cooling in the atmosphere and the extent to which water vapor facilitates a feedback between temperature and radiation. At face value, this sounds hopeless for a theoretician: Adding gray radiation (Planet 1 in Section 1) to a dynamical theory is tough enough; none of the dozen or so theories in the literature directly incorporate radiative transfer. And even if a clever dynamicist managed to do so, a qualitatively different answer would arise if one of the more sophisticated radiation schemes were used instead. Just accounting for a few bands of radiation (Planet 2), even without water vapor feedback, is sufficient to change the sign of the response.

But all is not lost; Tan et al. (2019) argue that the key impact of radiation may lie in how it determines the mean state of the atmosphere. While they took great care to ensure that global mean temperature and climatology were consistent across the four planets they consider—which was no small task—there were still fundamental differences in the atmospheric structure. The gray radiation scheme is associated with a so‐called split jet, where the subtropical jet (the baroclinic jet associated with the Hadley cell) is well separated from the eddy‐driven or midlatitude jet (which is associated with surface westerlies generated by eddy momentum fluxes). In the other configurations with a more realistic representation of radiative transfer, the subtropical and midlatitude jets were more merged together, as they are (generally) observed on Earth.

This insight provides us two paths forward. First, an accurate representation of the basic state is essential for climate prediction; continued effort to reduce biases in historical climate integrations—the arduous, and often not sufficiently rewarded work of model development—is vital. Second, for further theoretical development, mechanisms must explicitly account for the mean state. If a proposed mechanism cannot differentiate the response of the gray atmosphere from a more realistic configuration, it is not sufficient to help us explain the climate response to warming.

## 4 Connecting Theory to Comprehensive Atmospheric Models

The hierarchy of atmospheres explored by Tan et al. (2019) allowed them to highlight specific processes that are critical in the circulation response. Their results support a growing awareness that stratospheric temperature trends play a key role in tropospheric circulation trends (e.g., Manzini et al., 2014; Grise & Polvani, 2017). They also find that latitudinal extent of the warming response in the tropics plays a key role in the circulation response (cf. Tandon et al., 2013). This mirrors the insight we gain from El Niño–Southern Oscillation, where a warming of the tropics causes the extratropical jets to contract toward the pole (Seager et al., 2003), while global warming tends to push the jets apart (Lu et al., 2008).

Tan et al. (2019) developed a “diabatic hierarchy,” a sequence of models that are identical except for the their representation of the internal diabatic processes in the atmosphere (Maher et al., 2019). Beginning with the gray atmosphere developed by Frierson et al. (2006), they worked their way up to a representation of full radiation (albeit without clouds), similar to configurations established by Merlis et al. (2013) and Jucker and Gerber (2017). While the quantitative response of the jet depends critically on the radiative transfer scheme, Tan et al. (2019) suggest that fairly simple configurations, Planets 2 and 3, with only a few bands in the long wave and a simplistic water vapor feedback, may be sufficient to capture the qualitative response. They could be good targets for further theoretical development.

Model hierarchies help to build bridges between theoretical understanding of the atmospheric circulation and its representation in increasingly complex climate and weather prediction systems (Maher et al., 2019, and references therein). For example, we can connect the CO_2_ response of climate models all the way down to the very simple algebraic “layer models” of Arrhenius (1896), giving us greater confidence that we understand *why* our complex models are warming and which processes are essential (e.g., the water vapor feedback) and so deserving of detailed scrutiny. In comparison, the circulation response to anthropogenic forcing is harder to pin down at a basic process level. We are left describing what our models do, instead of evaluating the key drivers of the response in order to test them against what we can observe.

**Figure 2:**
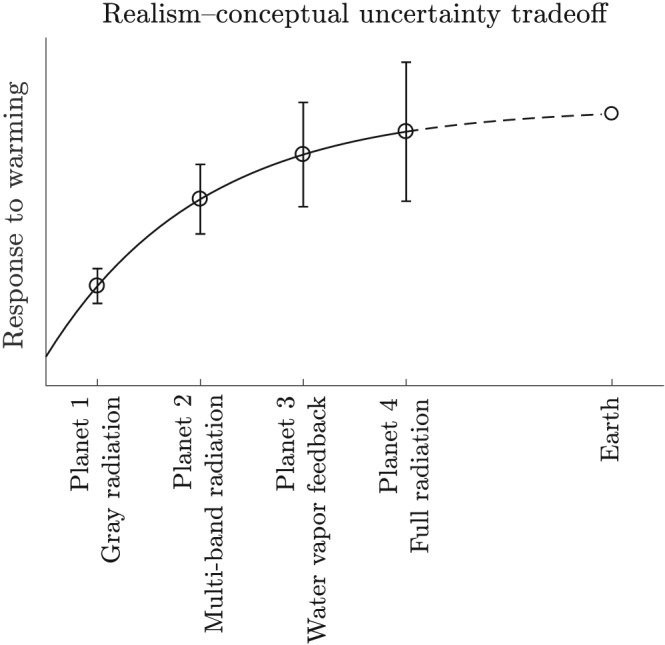
The tradeoff between conceptual uncertainty (i.e, the precision at which we understand the model) and realism (i.e., the accuracy of the forecast) in the model hierarchy of Tan et al. (2019). As the treatment of radiative transfer increases in realism, progressing from a gray atmosphere to full radiation (as found in a comprehensive atmospheric model), we expect the response to approach that of the true Earth. As the models increase in complexity, however, the conceptual uncertainty, represented by the error bars, also increases. While a most realistic representation of the Earth system is crucial for quantitative climate prediction, much can be learned by fully understanding simpler systems.

Reducing the conceptual complexity is a key element of a hierarchical approach, as illustrated in Figure 2. There were several significant simplifications in the atmospheres of Tan et al. (2019). One was to remove zonal asymmetries: Before we tackle differences in the North Atlantic and North Pacific jets (Figure 1), can we nail down a theory for one homogeneous jet? A second key simplification was to remove the influence of clouds: Before we introduce uncertainties due to parameterization of clouds, can we understand circulation feedbacks with clear sky radiation? Answering these questions for intentionally simplified planets increases confidence in state‐of‐the‐art model results.

The work by Tan et al. (2019) joins a growing number of efforts to build up a modeling infrastructure for the atmospheric circulation. Notably, the Isca framework of Vallis et al. (2018) allows one to construct and use similar diabatic hierarchies. Isca was developed within the Geophysical Fluid Dynamics Laboratory's Flexible Modeling System framework. The SimplER project within the Community Earth System Model framework is making available more idealized configurations of the National Center for Atmospheric Research's models (Polvani et al., 2017). In particular, these model hierarchies come with user support to both compile and configure models. When multiple groups focus on similar atmospheric models, there is a chance to reproduce key results, identify inconsistencies (and coding bugs), and push forward new questions.

## 5 Exploring Climate Regimes Beyond Our Own

While idealized models are sufficiently motivated by the need to study Earth's climate, they do provide a pathway to explore the circulation of exoplanets as well. For example, Kaspi and Showman (2015) explore a range of exoplanetary atmospheres with essentially the same gray atmosphere model used by Tan et al. (2019), Planet 1 in the sequence of section 1 and Figure 2, but over a much broader range of climatologies.

Advances in observational capacity are making this work less hypothetical. One example is the recent observations of Martian atmosphere used by Kahre et al. (2015) to assess radiative‐dynamic feedback due to observed dust and water cycles on Mars. Farther afield, Wolf (2017) uses an earlier version of Community Climate Model (CCM4) with a slightly modified radiation scheme to explore the range of habitability for exoplanets in the TRAPPIST‐1 system. With a sufficiently high concentration of CO_2_ (or other greenhouse gas), planet “e” (the fourth of seven planets orbiting the TRAPPIST‐1 dwarf star) could potentially sit within the range of habitability, warm enough to sustain liquid water but cool enough to avoid a runaway greenhouse effect.

## 6 Models of Simpler Atmospheres

The models explored by Tan et al. (2019) are generally referred to as idealized atmospheric models, or idealized general circulation models. We suggest a subtle but important rearrangement of the nomenclature. Rather than viewing them as *simple models of our atmosphere*, consider them as *models of simpler atmospheres*.

It is true that some idealized models are explicitly constructed to capture a simplified representation of the real atmosphere. In so‐called Earth systems Models of Intermediate Complexity (Claussen et al., 2002), the representation of the atmosphere, and all other components of the Earth system, is deliberately simplified to make them computationally efficient. Even our state‐of‐the‐art climate prediction models are highly idealized compared to the real atmosphere, although here the simplification is not deliberate but set by the limits of our understanding and computational power.

Tan et al. (2019) had a different aim. Their alternative atmospheres are well defined and deliberately simplified, to allow them to focus in on the role of a few key processes: radiative transfer and dynamics. Their models can be viewed as “recipes” for simpler atmospheres which could be explored independent of a given model framework. For example, their results could be tested across different resolutions, to ensure an accurate representation of the dynamics, or across different numerical representations of radiative transfer, for example, the model of Merlis et al. (2013) as compared to Jucker and Gerber (2017).

Held (2005) makes an analogy with biology, which has made great strides in understanding the human body by focusing large efforts on “simpler” creatures. While evolution has provided a hierarchy to biology, climate scientists must decide themselves if simpler “climate models of lasting value” can help us understand our Earth. Viewed through a dynamical lens, Tan et al. (2019) have posed a few simpler planets that could be studied with the aim of working our way up toward a full Earth's atmosphere.

To make full use of these model systems, we can increase their accessibility. Efforts to *meaningfully* share code, expertise, and development through freely available code repositories, such as in the Isca framework (Vallis et al., 2018), could make this possible. Not only does this make our science more accessible to the growing international research community but it also allows work on simplified atmospheres to progress toward an elegant and dynamically consistent hierarchy for understanding Earth's atmosphere.
